# A comparative biomechanical study of the Distal Tibia Nail against compression plating for the osteosynthesis of supramalleolar corrective osteotomies

**DOI:** 10.1038/s41598-021-97968-z

**Published:** 2021-09-22

**Authors:** Julia Greenfield, Philipp Appelmann, Yoann Lafon, Karine Bruyère-Garnier, Pol Maria Rommens, Sebastian Kuhn

**Affiliations:** 1 Univ Gustave Eiffel, IFSTTAR, LBMC, UMR_T9406, Univ Lyon, 25 Avenue François Mitterrand, 69500 Bron, France; 2 Department of Orthopaedics and Traumatology, University Medical Centre of the Johannes Gutenberg University, Langenbeckstrasse 1, 55131 Mainz, Germany; 3 Department of Digital Medicine, Medical Faculty OWL, Bielefeld University, Universitätsstr. 25, 33615 Bielefeld, Germany

**Keywords:** Medical research, Engineering

## Abstract

The Distal Tibia Nail (DTN; Mizuho, Japan) has demonstrated higher biomechanical stiffness to locking plates in previous research for A3 distal tibia fractures. It is here investigated as a fixation option for supramalleolar corrective osteotomies (SMOT). Sixteen Sawbones tibiae were implanted with either a DTN (n = 8) or Medial Distal Tibia Plate (MDTP; n = 8) and a SMOT simulated. Two surgical outcome scenarios were envisaged: “best-case” representing an intact lateral cortex, and “worst-case” representing a fractured lateral cortex. All samples were subjected to compressive (350 N, 700 N) and torsional (± 4 Nm, ± 8 Nm) testing. Samples were evaluated using calculated construct stiffness from force–displacement data, interfragmentary movement and Von Mises’ strain distribution. The DTN demonstrated a greater compressive stiffness for the best-case surgical scenario, whereas the MDTP showed higher stiffness (*p* < 0.05) for the worst-case surgical scenario. In torsional testing, the DTN proved more resistant to torsion in the worst-case surgical setup *(p* < 0.05) for both ± 4 Nm and ± 8 Nm. The equivalent stiffness of the DTN against the MDTP supports the use of this implant for SMOT fixation and should be considered as a treatment option particularly in patients presenting vascularisation problems where the MDTP is an inappropriate choice.

## Introduction

Lower limb malalignment is a frequent post-operative condition due to the operative procedure or malunion; however, this problem can also be related to genetic disposition, as is the case in people with bowed legs as a birth deformity^1^. Joint malalignment leads to an imbalance in load transmission across the joint surface resulting in increased impact and compressive loads on a part of the joint^2,3^. Individuals with a joint malalignment are at greater risk of osteoarthritis (OA)^4–6^. Corrective osteotomies are carried out to straighten the axis of a long bone in order to improve load transfer across the joint surface^2^. In the case of the distal tibia, a SupraMalleolar OsteoTomy (SMOT) is carried out to realign the ankle joint where the tibial pilon meets the superior surface of the talus bone^7,8^. Following this procedure, an implant is used to stabilise the osteotomy; to date the only available implants for this procedure are locking or dynamic compression plates^9^. The most important disadvantage of plate osteosynthesis at the distal tibia is the need for a large medial incision, which may be the origin of wound healing disturbances and infection. Especially in patients with compromised soft tissue coverage, the risk of complications is enhanced^10^.

Previous research for SMOT has focused on the evaluation of the osteotomy zone^9,11^ and changes in plantar pressure zones following osteotomy^12^. Ettinger et al.^9^ is the only study to have carried out biomechanical assessments of the different compression plates available for SMOT, finding a difference of up to 632 N mm^−1^ in axial stiffness (range 1985–2617 N mm^−1^) and 0.66 Nm deg^−1^ in torsion stiffness (range 3.48–4.14 Nm deg^−1^) between models.

The Distal Tibia Nail (DTN; Mizuho, Japan) is a new treatment option for far distal fractures, covering the zone in which a SMOT would be performed^10,13,14^. Plating has been a solid option until now but patients with already compromised soft tissue in the distal tibia are put at risk when using this method^14–16^. The aim of this study was to assess the biomechanical stiffness of the DTN compared to the current standard fixation method of plating for SMOT fixation. The assumption is made that if the DTN is not significantly lower in stiffness than the Medial Distal Tibia Plate, then it can be considered as an intramedullary fixation option for SMOT. Based on results from previous studies^10,13,14^, we hypothesise that the DTN will demonstrate a greater biomechanical stiffness compared to the plate.

## Materials and methods

**Figure 1:**
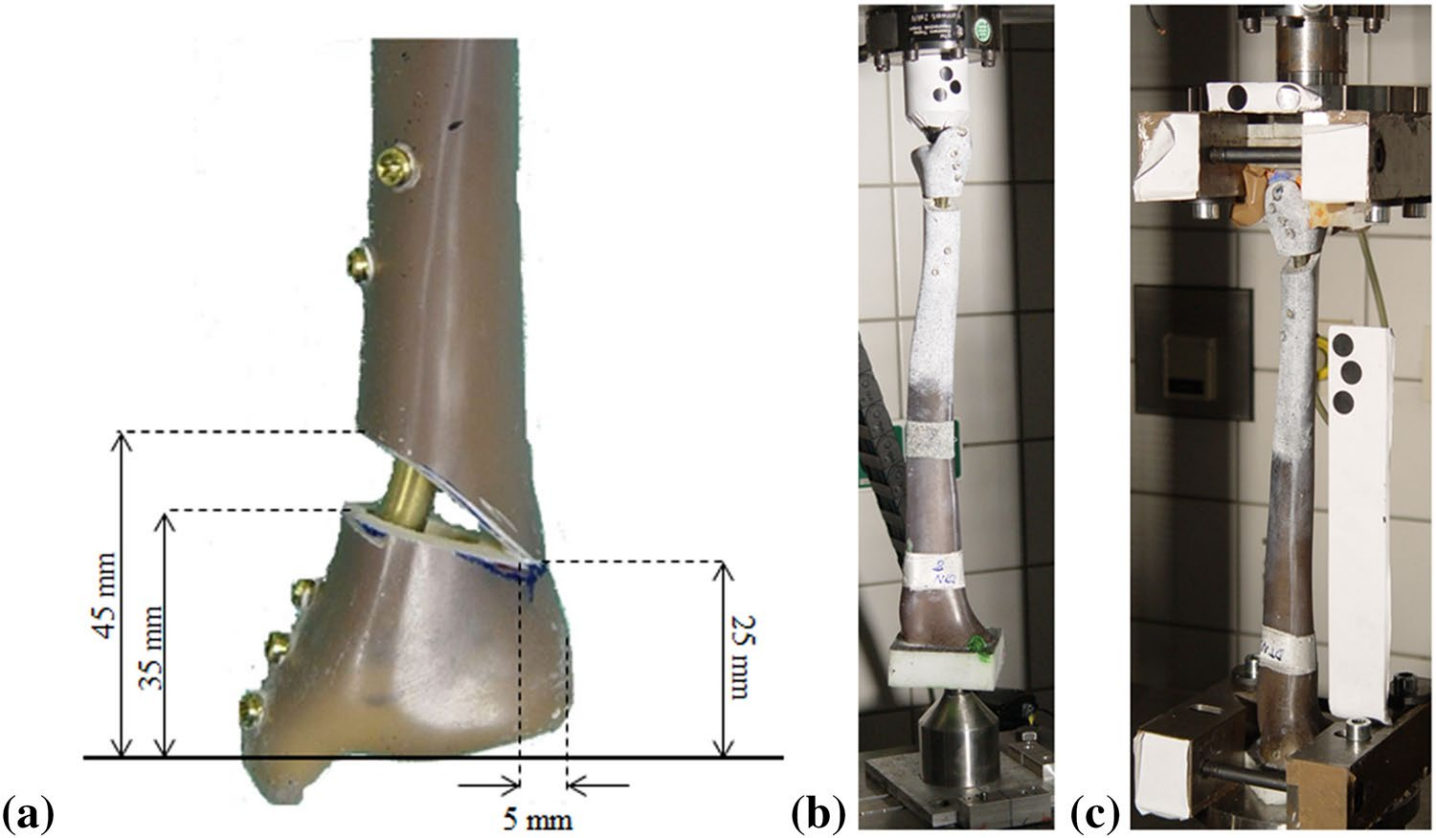
Planned measurements for the medial wedge osteotomy simulation used in this study (**a**), and test setups for compressive (**b**) and torsional (**c**) testing.

Sixteen Sawbones (left-side, medium size, item #3401, Malmö, Sweden) were implanted with a Medial Distal Tibial Plate (MDTP; Synthes, Switzerland; n = 8) or a DTN (n = 8) and a Medial Wedge Opening (MWO) osteotomy simulated at 45 mm proximal from the distal articular tibial surface. A brief description of the DTN and MDTP implantation procedures can be found in Kuhn et al.^14^ and AO Foundation surgery reference^17^, respectively. Screw insertion for both implants is conducted through small incisions in the skin using a scalpel. The wedge was 10 mm in height and left 5 mm of lateral cortex intact (Fig. 1a). This was known as “best-case scenario” SMOT where the lateral cortex is fully intact. Following a first round of biomechanical testing (explained below), “worst-case scenario” SMOT was simulated where the lateral cortex of all samples was cut using a 0.5 mm coping saw. This latter phase replicated a fracture of the lateral cortex, which can occur during the surgical procedure. In true surgical situations, the distal tibia is prized open to create a wedge-shaped gap; in our study, a wedge of composite bone was cut from the distal tibia using a manual saw. The fibula was omitted from all tests due to complications relating to the inclusion of an inter-osseous membrane connecting the tibia and fibula^18^. In vivo, this membrane is primordial for the force transmission between the tibia and fibula, however no composite structure to date has been developed to replicate this phenomenon.

All samples were proximally embedded in polymethyl methacrylate (PMMA, Swiss Composite, Switzerland) using a template leaving a thickness of < 2 mm of PMMA at the loading site. For digital image correlation (DIC) analysis, two cameras (JAI-GO 5000 USB, JAI A/S, Grosswallstadt, Germany; full resolution 2474 × 2076 pixels) were placed facing the sample at a divergence of 16°. All samples were covered with white paint and a black speckle pattern (not affecting the mechanical behaviour of the underlying materials). DIC allows for a non-invasive, contactless technique for the measurement of displacement and strain fields across a surface^19^. A review of this method can be found in Rankin et al.^20^.

### Biomechanical testing

All samples were subjected to extra-axial compression tests^13,14^ of 350 N at 0.1 Hz followed by 700 N at 0.05 Hz with an 18 N compressive pre-load applied. For compression testing, the samples were placed in a double ball-joint setup (Fig. 1b). The proximal loading point was considered to be at the physiological loading axis taking into account the 60/40% medio-lateral load distribution across the tibial plateau^21–23^, located at 10 mm medio-posterior to the central axis, generating a lever arm of 14.14 mm. The distal ball-joint was placed in the central axis of the Sawbones samples. Following this, torsional tests of ± 4 Nm at 0.1 Hz, and ± 8 Nm at 0.05 Hz were performed, no torsional pre-load was applied to the samples; however, a 6 N compressive load was applied throughout all torsional testing to ensure sample stability in the test setup. Torsion tests were carried out by proximally and distally clamping the sample in the testing machine. The proximal PMMA was set in the inferior joints and the distal end of the sample was clamped in a detachable PMMA block in the superior joints (Fig. 1c). All tests were conducted over 30 cycles. Fatigue testing at 1000 + cycles was not chosen for this study as the aim was to carry out a preliminary investigation into the bone-implant construct behaviour. Machine data was recorded at 25 Hz, still camera images were recorded at 10 Hz. The machine and camera data acquisitions were synchronised using a trigger sent by the testing machine at the start of testing.

### Stiffness evaluation

**Figure 2:**
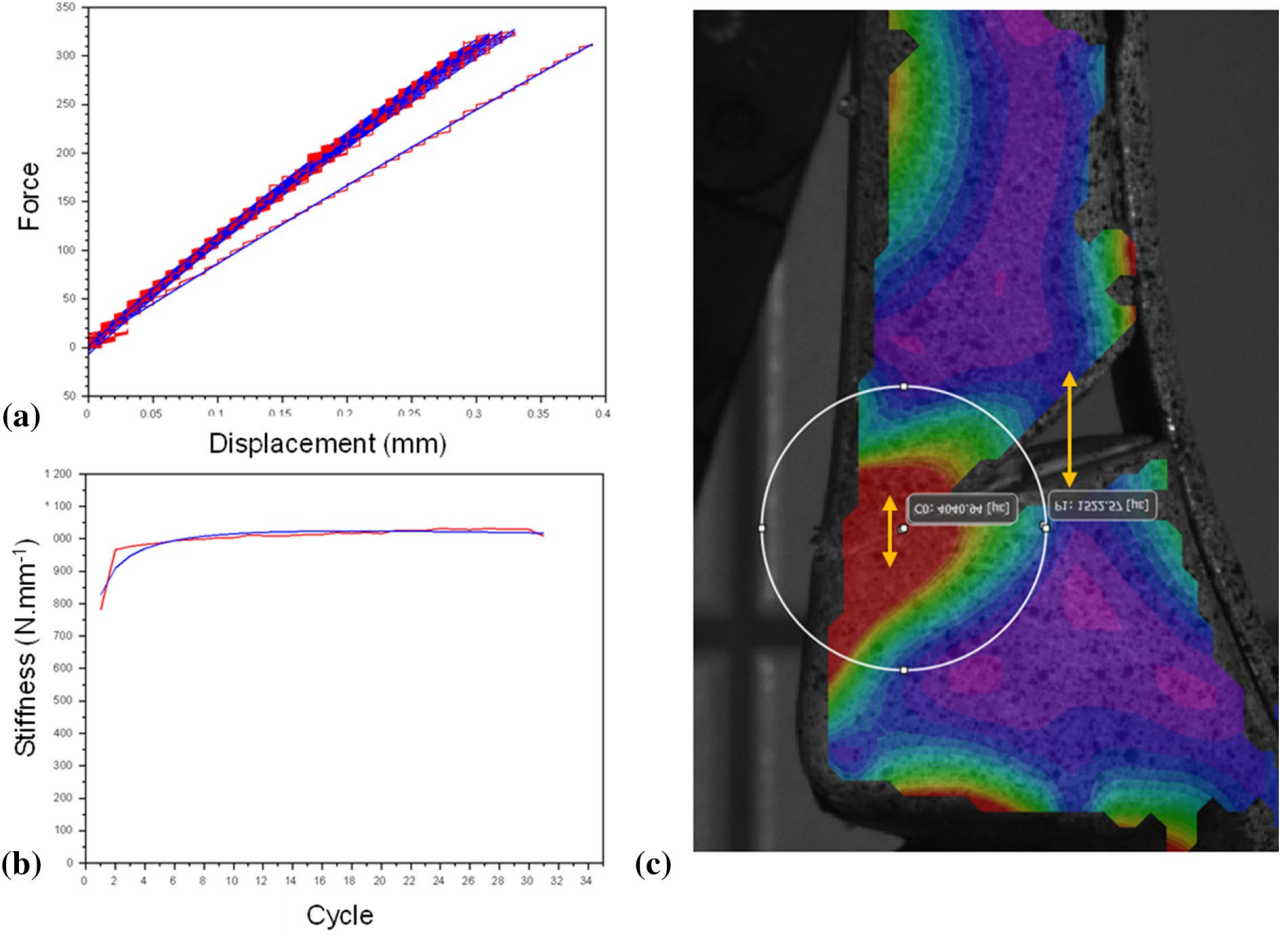
Force–displacement (**a**) and stiffness (**b**) curves for a given sample. The red line indicates raw data while the blue line indicates interpolated data using a 2nd order polynomial trend-line. Extensometer (yellow arrows) and nodal disc outline (white circle) placements for measurement of osteotomy gap displacement and strain due to extra-axial compression (**c**); red/warm colours indicate high Von Mises’ strain, blue/cold colours indicate low Von Mises’ strain. The example strain image was obtained from Vic3D software (version 8), https://www.correlatedsolutions.com/vic-3d/.

Sample construct stiffness were calculated using an in-house code written in Scilab (version 6.0.2). During preliminary testing, lower construct stiffness detected in the first 5 cycles due to the bedding in of the test setup (Fig. 2a,b) 30 cycles lead to the achievement of a stable stiffness evaluation. The erroneous data in the final cycle was put down to false detection of the end of the cycle and the force actuator returning to its initial position. For these reasons, only cycles 6–28 were considered in the construct stiffness calculation so to achieve consistent results. Stiffness was calculated by plotting all the points of the force–displacement (or torque–angle) testing machine data and taking the Theil-Sen estimator^24^ of the slope between adjacent points. Stiffness was calculated for all compression testing at 200 ± 100 N (remaining in the linear elastic deformation zone), and for torsional testing at 2.5 ± 0.5 Nm for the ± 4 Nm tests and 6.5 ± 1 Nm for the ± 8 Nm tests. Mean construct stiffness ± two standard deviations are cited as well as the range (minimum to maximum stiffness).

### Interfragmentary movement and strain evaluation

Image data were processed in Vic3D software (version 8, Correlated Solutions, Inc, USA). The facet and step sizes used for the correlation were 25 and 7 mm, respectively displacement uncertainty (considered as 1/100 of pixel size) was 0.003 mm.

The image corresponding to the maximum force application of the last complete compression cycle was identified, and then used to calculate average interfragmentary movement (IFM) in two zones, the lateral cortex and the osteotomy gap, using the Vic3D extensometer tool (Fig. 2c).Von Mises’ strain was exported from a zone corresponding to a nodal disc placed around the osteotomy site, with the disc centre being at the midpoint between the narrowest part of the osteotomy and the lateral cortex; all nodal discs had a radius of 15 mm giving around 350 measured nodes exported. The average maximal Von Mises’ strain value corresponded to the mean of the highest 10% of nodal strain values; this latter step was performed to privilege the comparison of the maximum strain levels in this area, for the reduction of the disc positioning uncertainty. DIC was only carried out for compression testing at 700 N as it was not possible to correlate the MDTP during torsional testing due to the camera angle falling on the uneven surface of the plate.

### Statistics

Statistical tests were undertaken in Statgraphics Centurion 18 (version 18.1.09, Statgraphics Technologies, Inc., Virginia, USA). Tests for normality and heteroscedasticity were carried out using the Shapiro–Wilk and Levene’s tests, respectively. The DTN (n = 8) and MDTP (n = 8) samples were treated as independent sample groups. Biomechanical stiffness constructs were compared between sample groups and within a specific testing phase (best or worst –case surgical scenario) using the Student’s t-test for data presenting a normal distribution and the Mann Whitney-U test for non-normal data.

## Results

All results are presented in graphic form displaying the mean stiffness ± two standard deviations for comparison of the DTN and MDTP implanted samples for all applied loads. A summary table of all main results is given at the end of the stiffness section (Table 1).

**Table 1:** Average compression and torsional construct stiffness ± two standard deviations for all samples based on applied load and implant.

Surgical scenario	Load	Implant	Mean stiffness ± 2SD	Range
			N mm^−1^ or Nm deg^−1^	
Best-case scenario, intact lateral cortex	− 350 N	DTN	1311 ± 10	1010–1734
		MDTP	1287 ± 11	860–1689
	− 700 N	DTN	1332 ± 10	968–1641
		MDTP	1250 ± 9	943–1250
	± 4 Nm*	DTN	2.74 ± 0.06	2.27–3.24
		MDTP	3.47 ± 0.07	2.95–3.88
	± 8 Nm	DTN	2.18 ± 0.04	1.80–2.51
		MDTP	2.06 ± 0.06	1.38–3.01
Worst-case scenario, fractured lateral cortex	− 350 N*	DTN	903 ± 9	790–1064
		MDTP	1162 ± 12	721–1353
	− 700 N*	DTN	957 ± 22	778–1095
		MDTP	1193 ± 10	913–1392
	± 4 Nm*	DTN	0.96 ± 0.30	0.63–1.23
		MDTP	0.71 ± 0.12	0.45–0.85
	± 8 Nm*	DTN	1.42 ± 0.04	1.15–1.76
		MDTP	0.65 ± 0.07	0.50–0.99

The range is given based on minimum to maximum construct stiffness.

SD, standard deviation.

*Significant difference at the 95% level between the DTN and MDTP implant groups.

### Compressive stiffness

**Figure 3:**
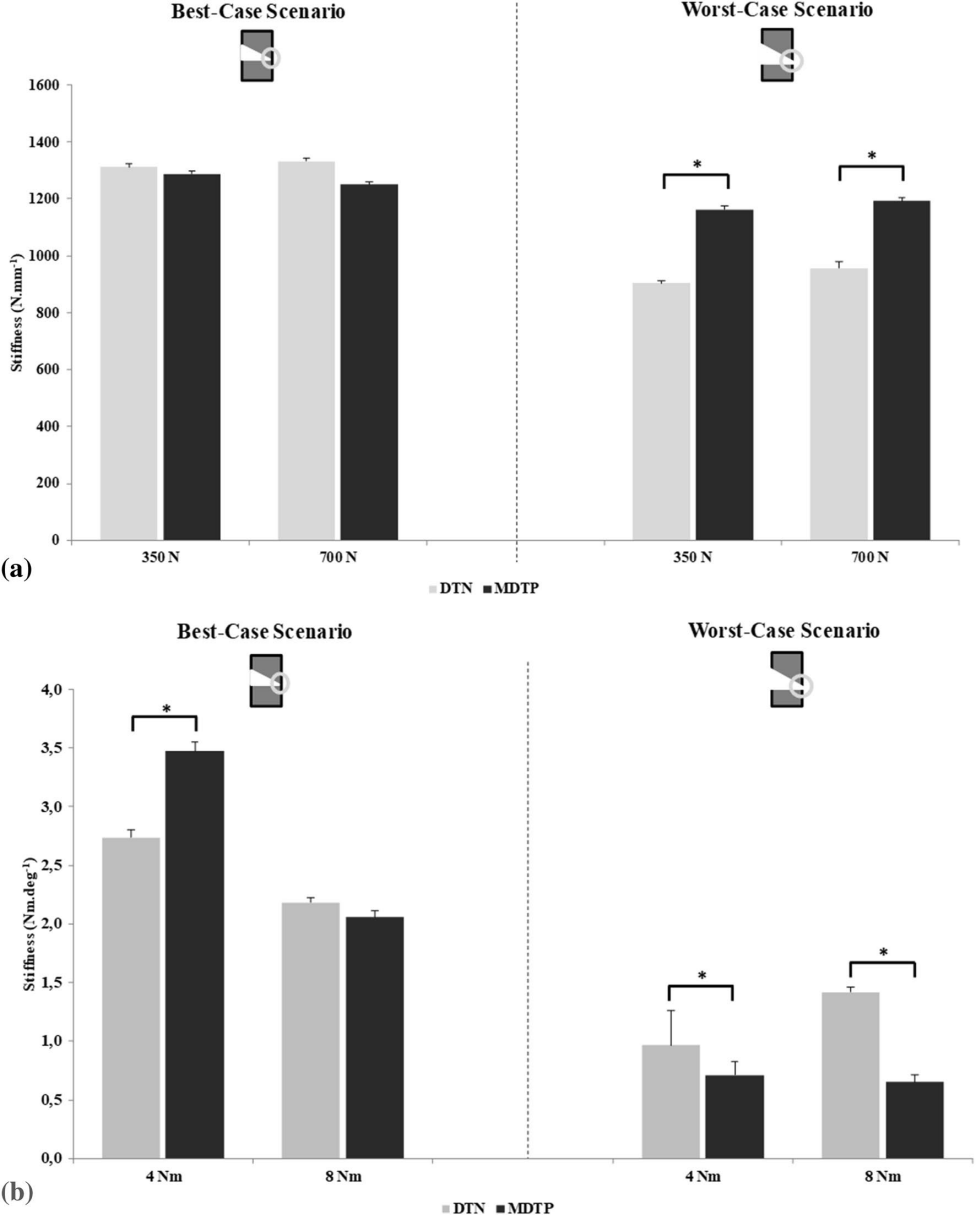
Mean ± 2SD compression stiffness (**a**) and torsional stiffness (**b**) results for the two surgical scenario (n = 8 samples for each implant group) and two loading levels. Bars marked with *indicate a significant difference between the implant groups.

The samples’ average construct stiffness for the best-case surgical scenario are similar with marginally higher stiffness for the DTN group during compression testing (Fig. 3). At 350 N, mean construct stiffness were measured to be 1311 ± 10 N mm^−1^ for the DTN group, and 1287 ± 11 N mm^−1^ for the MDTP implanted group. At 700 N, there was a greater difference between the groups (DTN: 1332 ± 10 N mm^−1^, MDTP: 1250 ± 9 N mm^−1^; however, no significant differences were observed between groups.

For the worst-case surgical scenario, at 350 N, there was a significant difference between implanted groups with the MDTP demonstrating higher levels of compressive stiffness (1162 ± 12 N mm^−1^ against 903 ± 9 N mm^−1^; U = 56, *p* = 0.01). A significant difference between implant groups was also observed for 700 N compression test (1193 ± 10 N mm^−1^ vs 957 ± 22 N mm^−1^ for the MDTP and DTN, respectively; t (14) = -3.87, *p* = 0.002).

### Torsional stiffness

**Figure 4:**
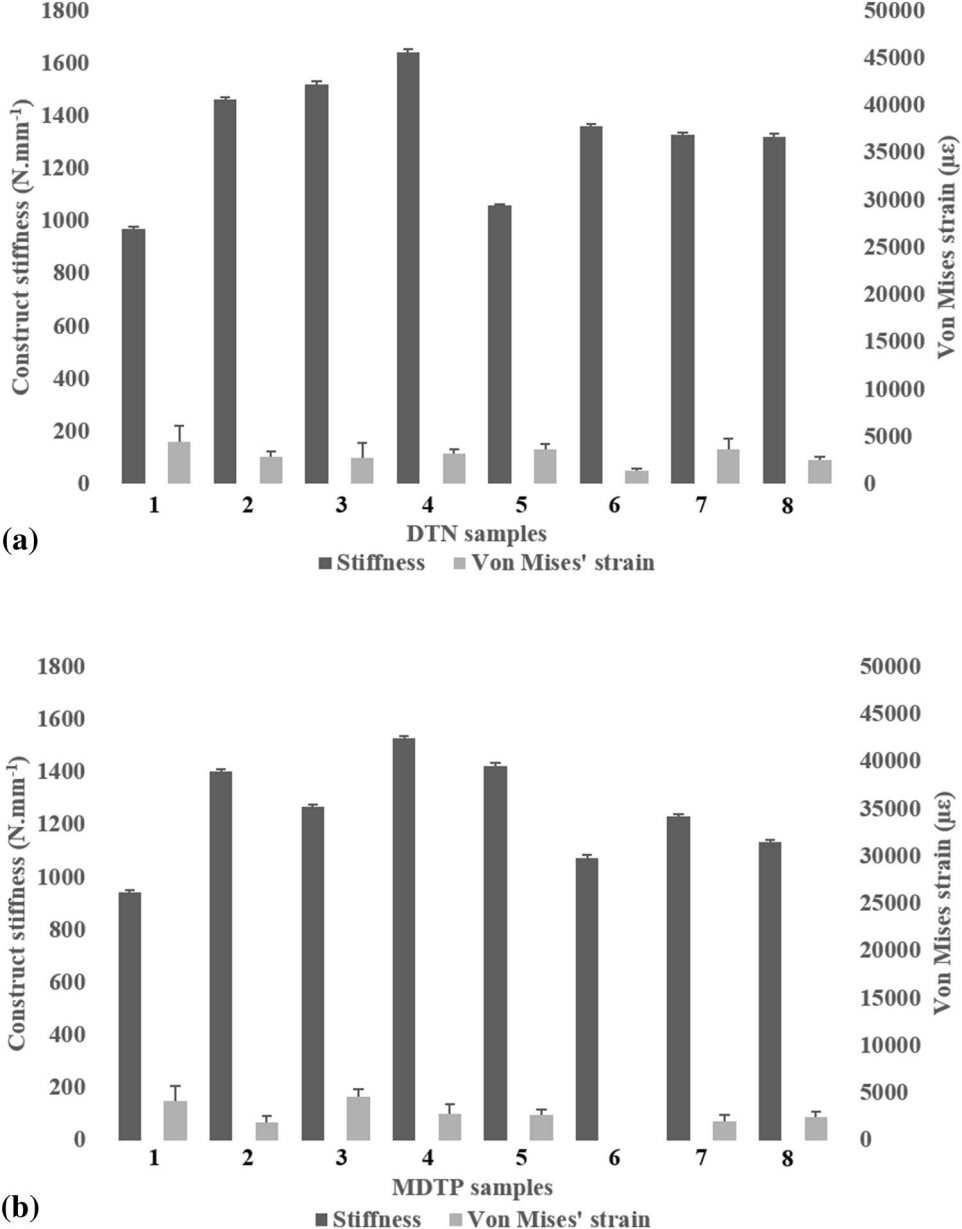
Average maximum Von Mises strain ± 2 standard deviations, and mean construct stiffness ± 2 standard deviations with strain distribution images for the DTN (top) and MDTP (bottom) samples at 700 N compressive loading in the best-case surgical scenario. For the MDTP samples, n = 7 as the MDTP-6 sample is missing for this set of tests due to corrupt data.

For best-case scenario testing (Fig. 4), mean torsional stiffness during ± 4 Nm testing was at 2.74 ± 0.06 Nm deg^−1^ for the DTN, and significantly higher for the MDTP group at 3.47 ± 0.07 Nm deg^−1^ (t(14) = − 4.51, *p* < 0.01; Fig. 4). At ± 8 Nm, data was found to deviate from normality and therefore a Mann–Whitney U test was applied. No significant differences were found between the sample groups with the DTN group presenting a mean stiffness of 2.18 ± 0.04 Nm deg^−1^ and the MDTP group 2.06 ± 0.06 Nm deg^−1^.

In worst-case scenario testing, a Mann Whitney-U test found the DTN to have a greater torsional stiffness at ± 4 Nm (0.96 ± 0.30 Nm deg^−1^ against 0.71 ± 0.12 Nm deg^−1^, for the DTN and MDTP, respectively; U = 11, *p* = 0.03). At ± 8 Nm, the disparity between the construct stiffness of the two implants was greater with values of 1.42 ± 0.04 Nm deg^−1^ against 0.65 ± 0.07 Nm deg^−1^, for the DTN and MDTP, respectively (U = 0; *p* < 0.01).

### Digital image correlation

**Figure 5:**
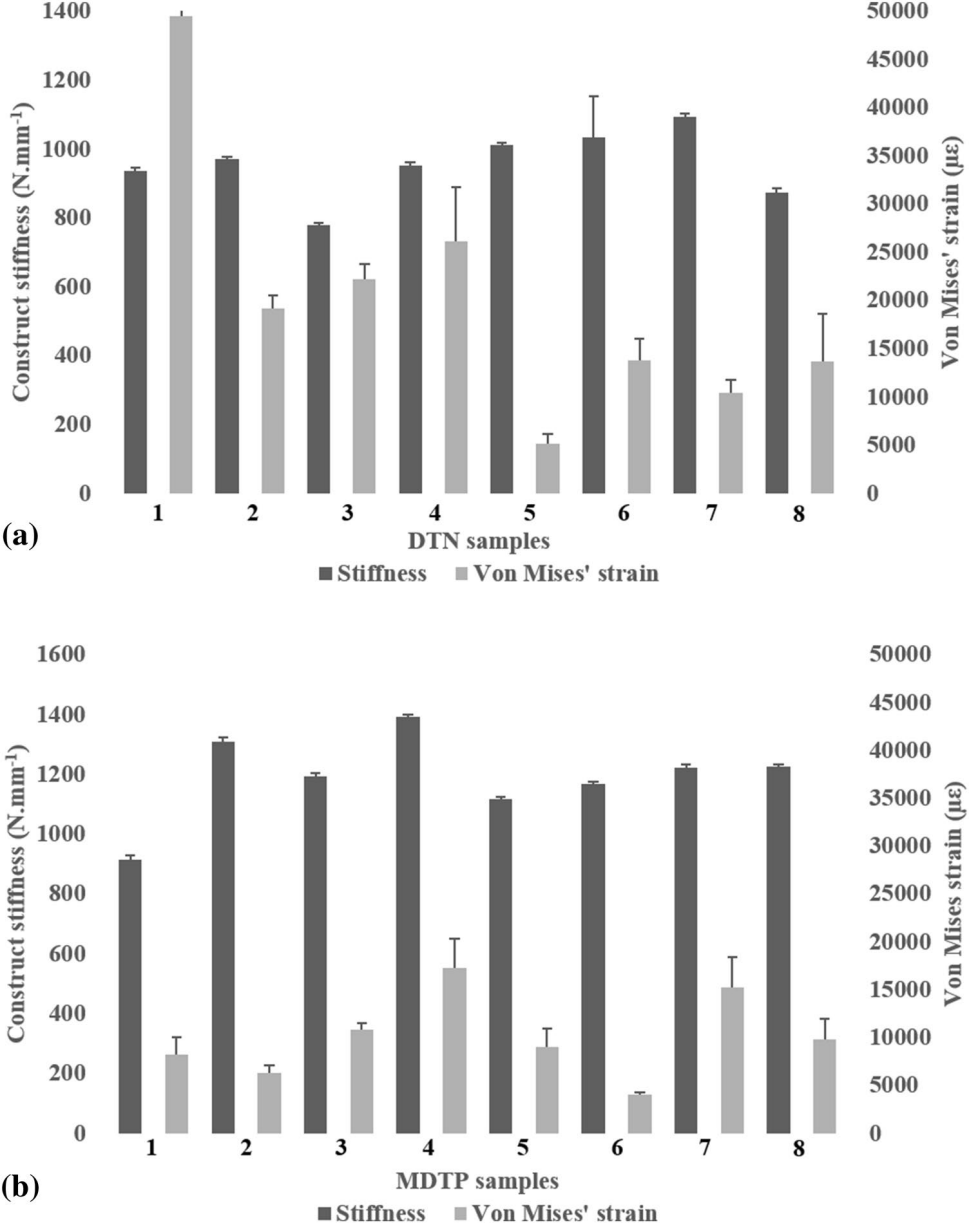
Average maximum Von Mises strain ± 2 standard deviations, and mean construct stiffness ± 2 standard deviations with strain distribution images for the DTN (top) and MDTP (bottom) samples at 700 N compressive loading in the worst-case surgical scenario. Note that the error bar representing standard deviation for the DTN sample 1’s average maximum Von Mises strain is out of the chosen scale (49,502 ± 6194 µε).

Average maximal Von Mises’ strains around the osteotomy and mean construct stiffness are plotted against each other in Figs. 4 and 5 for best and worst –case surgical scenario, respectively. Generally, an increase in stiffness is associated with lower average maximal Von Mises’ strain values around the osteotomy for the DTN (Fig. 4a) and the MDTP (Fig. 4b) samples; however, this is not a set rule and strain values vary, regardless of the construct stiffness.

In the best-case scenario, average maximal Von Mises’s strains at the osteotomy site are quite similar across all samples. In the worst-case surgical scenario, a sharp increase in strain at the osteotomy site is witness across all samples, despite a relatively low decrease in stiffness construct (Fig. 5). Very high Von Mises’ strain is observed in the DTN-1 sample (Fig. 5a) at an average of 49,502 ± 6194 µε. In order to preserve identical scales for all graphs, the y-axis is cut at 30,000 µε. No trend is seen between construct stiffness and average maximal strain. On closer observation of the samples, it appeared that shear movement between the two parts of the bone occurred, particularly in the DTN samples, witnessed once the osteotomy had been completed.

Average maximal Von Mises’s strain around the osteotomy (ε_VM_) and IFM are reported in Table 2. A best-case surgical outcome scenario yielded no significant differences both for ε_vm_ and IFM_mid_ (*p* = 0.06), however IFM_lat_ yielded significant differences (*p* < 0.05) between the DTN and MDTP implanted samples (Table 2). Average maximal Von Mises’s strain around the osteotomy in the DTN sample group was 3066 ± 1823 µε (range 1432–4492 µε), and 2933 ± 2053 µε (range 1914–4575 µε) for the MDTP samples. In the worst-case surgical scenario, the Von Mises’s strain around the osteotomy measured in the DTN samples averages at 20,023 ± 27,268 µε, and ranges from 5193 to 49,502 µε; whereas the range in the MDTP samples is less pronounced and lower, averaging at 10,107 ± 8789, and ranging from 4052 to 17,316 µε. The worst-case surgical scenario produced significantly higher IFM for the DTN implanted group (*p* = 0.01), both across the fracture gap and the lateral cortex area; no statistically significant difference was observed for the average maximal Von Mises’ strain (*p* = 0.06).

**Table 2:** Average Maximal Von Mises’ strain around the osteotomy (ε_VM_) and interfragmentary movement (IFM) for all DTN (n = 8) and MDTP (n = 8) samples in a best and worst-case surgical outcome setting and a compressive load of 700 N.

Surgical scenario	Parameter	Implant	Mean ± 2SD
Best-case scenario, intact lateral cortex	ε_VM_ (µε)	DTN	3066 ± 1823
		MDTP**	2933 ± 2053
	IFM_mid_ (mm)*	DTN	− 0.15 ± 0.13
		MDTP	− 0.05 ± 0.00
	IFM_lat_ (mm)	DTN	− 0.013 ± 0.11
		MDTP	− 0.01 ± 0.01
Worst-case scenario, fractured lateral cortex	ε_VM_ (µε)	DTN	20,023 ± 27,268
		MDTP	10,107 ± 8789
	IFM_mid_ (mm)*	DTN	− 0.43 ± 0.26
		MDTP	− 0.10 ± 0.05
	IFM_lat_ (mm)*	DTN	− 0.35 ± 0.20
		MDTP	− 0.05 ± 0.04

ε_VM_, average maximal Von Mises’ strain around the osteotomy; µε, micro-strain; IFM_mid_, interfragmentary movement across the osteotomy gap; IFM_lat_, interfragmentary movement across the lateral cortex; SD, standard deviation.

*Significant difference at the 95% level between the DTN and MDTP implant groups.

**n = 7, data from the MDTP-6 sample is missing due to corrupt data.

**Figure 6:**
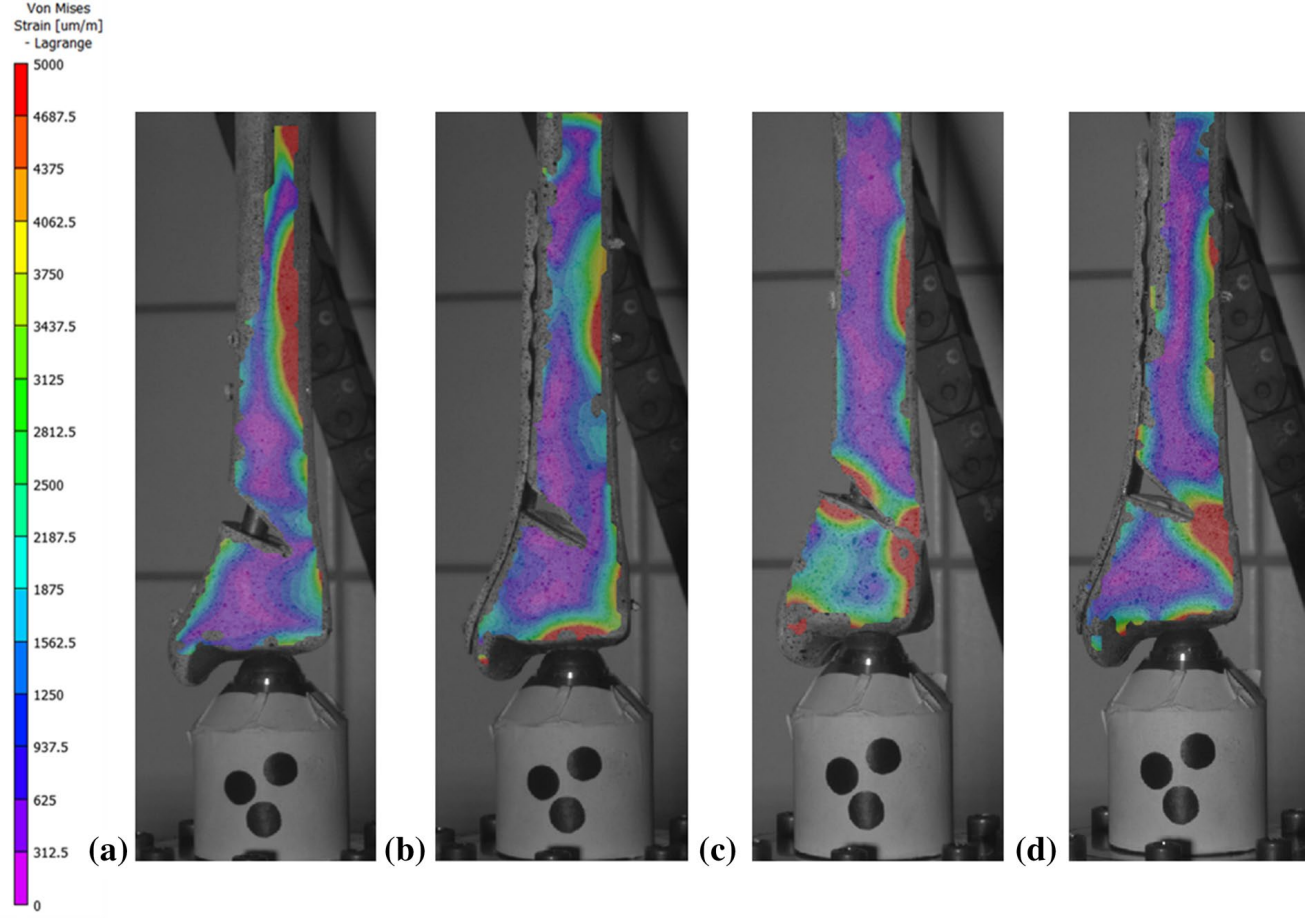
Von Mises’ strain (micro-strain) distribution for DTN (**a**, **c**) and MDTP (**b**, **d**) samples of similar stiffness in the best (**a**, **b**) and worst (**c**, **d**)-case surgical scenario; red/warm colours indicate high Von Mises’ strain, blue/cold colours indicate low Von Mises’ strain. Images correspond to samples DTN-3 (**a**), MDTP-4 (**b**), DTN-8 (**c**), MDTP-5 (**d**). Image obtained used Vic3D software (version 8), https://www.correlatedsolutions.com/vic-3d/.

The images presented in Fig. 6 correspond to one DTN (sample 3, stiffness = 1521 N mm^−1^; Fig. 6a) and one MDTP (sample 4, stiffness = 1529 N mm^−1^; Fig. 6b) sample presenting a similar stiffness construct for the best-case surgical outcome. For the worst-case surgical scenario, samples DTN-7 (stiffness = 1095 N mm^−1^; Fig. 6c) and MDTP-5 (stiffness = 1118 N mm^−1^; Fig. 6d) are shown.

The average maximal Von Mises’ strain distribution in the DTN (Fig. 6a) and MDTP (Fig. 6b) samples for the best-case surgical scenario demonstrate relatively low levels around the osteotomy site, but with more concentrated zones appearing on the lateral diaphysis and epiphysis. In the worst-case surgical scenario, greater levels of strain are seen around the osteotomy site, high levels in the lateral diaphysis remain present. In the DTN sample (Fig. 6c), an area of high strain can be observed at the medial opening of the osteotomy, close to where the nail passes. In the MDTP sample (Fig. 6d), two smaller zones of high strain are seen on the medial diaphysis and may be related to screw insertion sites.

## Discussion

The present study compared the DTN to the MDTP in compressive and torsional testing for supramalleolar osteotomy fixation. Construct stiffness of both methods is similar for the best-case surgical scenario, indicating that both methods are adequate for SMOT fixation where an intact osteotomy can be assured. In the worst-case scenario (involving a lateral cortex fracture), the DTN proved to be significantly lower in stiffness, leading us to reject our hypothesis. However, IFM levels do not exceed 1 mm for either of the implants; maximum difference in IFM was 0.33 mm across the osteotomy gap in the worst-case scenario for a compressive loading of 700 N. Hence, despite hypothesis rejection, low IFM levels support the use of the DTN for SMOT fixation. Where similar DTN and MDTP sample construct stiffness are observed, Von Mises’s strain levels are of equal magnitude in both the best- and worst –case surgical scenarios.

Ettinger et al.^9^ are the only authors to have conducted biomechanical tests on implants used for MWO in-situ. The MDTP was one of the implants (fixed to Sawbones samples) used in their study and the authors reported axial construct stiffness of 2182 N mm^−1^ (2182 kN mm^−1^ is reported in their article but this is an error of units used), compared to 1641 N mm^−1^—the maximum calculated stiffness value in the present study. The differing boundary conditions between the two test setups of the present study and that conducted by Ettinger et al. may explain the disparity in results. The present study employed ball joints allowing for axial rotation at the extremity of the sample. Ettinger et al. employed cardan joints which ensure degrees of freedom in rotation as us, but the centre of rotation is not identical regarding the tibial medial axis, leading to different moments. Further, during the present study, vertical displacement measured by the testing machine was quantified to be an overestimation of around 10% of the displacement measured by marker tracking. However, even after correction of this figure, our results are still not in line with those cited by Ettinger et al.

Images from Ettinger et al. give rise to the idea that substantial extremal potting of the samples was carried out, leading to principal testing of the tibial shaft, which is shorter than the tibia itself and the strongest part of the long bone (related to cortical thickness)^24^. In the current study, we ensured that no greater than 2 mm of PMMA was present between the tibial plateau and the universal joint through which compressive forces were applied. The distal potting could partially cover the distal implants as figured by Ettinger et al., and so influence the mechanical behaviour especially for the DTN. Furthermore, Ettinger et al. controlled for the amount of lateral cortex remaining post-osteotomy and set this to be 5 mm. In our study, the lateral cortex area was not regulated but it is estimated to be less than 5 mm for best-case scenario SMOT outcomes. Nonetheless, the figure reported by Ettinger et al. falls into a similar order of magnitude as in the current study.

Torsional stiffness is greater in the DTN samples for most test configurations—being more valuable in preventing shear strain. Shear movements are known to be harmful to bone remodelling if they exceed axial strain levels^2,5^. The significantly higher torsional stiffness presented by the MDTP sample group in the best-case surgical scenario at ± 4 Nm may be attributed to the low moment applied and the symmetry around the osteotomy gap. The in-tact lateral cortex and the medially positioned MDTP create a very stable structure allowing for little movement.

Measured torsional stiffness by Ettinger et al. agrees with data presented in the current study (Ettinger: 3.53 Nm deg^−1^ for loading at 0.25 Hz to 5 Nm; present study: 3.64 Nm deg^−1^ for loading at 0.1 Hz to 4 Nm). In this scenario, the difference in loading frequency (Ettinger: 2.5 Nm s^−1^; current study: 0.8 Nm s^−1^) did not appear to affect recorded stiffness values, as expected. These results are also in agreement with a previous study comparing the DTN to the MDTP, but for 43-A3 fractures^7^. The current article found to be 1.42 and 0.65 Nm deg^−1^ for the DTN and MDTP, respectively, during worst-case scenario testing (closely representing an A3 fracture). Kuhn et al. found 1.83 and 0.55 Nm deg^−1^, respectively^10^.

One may expect the DTN to present lower torsional stiffness due to its central positioning in the Sawbones sample. The higher construct stiffness recorded in the present study may relate to the nail’s position in the medullar cavity and the position of the screws within the nail and the 0.8 mm gap left from the difference in screw and screw hole–diameter (3.2 mm and 4 mm, respectively). A screw placed against the medial or lateral border of the screw hole will result in (for example) high positive torsional stiffness. Use of medical imaging technique would allow for the quantification of screw position with the designated hole. Such techniques were not employed during this study but should be taken into consideration in future analyses.

Recorded Von Mises’ strains fall into the category where new bone generation would be expected^25–27^. The difference in the average maximal Von Mises’ strains around the osteotomy site between the DTN and the MDTP implant groups for the worst-case scenario surgical outcome is high, but comparison with this level of strain in the literature suggests that this would not have a negative impact on the bone; however, this also depends on load frequency^28^. Quantification of Von Mises’ strain presented here must, however, be treated with caution as the edge effects will have influenced the mean strain quantified.

Lower stiffness is generally associated with higher Von Mises’ strain for best-case scenario sample groups, this may be also directly related to IFM. However, in some cases a sample with low construct stiffness also yielded low average strain; this discrepancy may be linked to the area over which these parameters are calculated. Stiffness calculations take into account the entire sample, whereas Von Mises’ strain calculations are only taken around the osteotomy site. External factors, such as machine setup and PMMA stiffness will influence total calculated stiffness but not strain distribution.

The greater and more concentrated strain around the osteotomy site in the worst-case scenario samples is likely due to the completion of the osteotomy cut through the lateral cortex. This detachment of the proximal and distal fragments allows for greater movement between the two. In the case of the DTN, this may increase bending motion during the compression testing, explaining the high strain zone at the osteotomy opening on the medial side. During compression, lateral bending may occur due to the extra-axial loading point and the proximal and distal fragments will push against the nail in the medial opening. High variability in the Von Mises’ strain of the DTN samples may owe to implant position inside the medullar cavity with or without contact with the cortex.

Maintaining an osteotomy gap can be associated to an A3 fracture for which the MDTP stiffness in consistently lower than that of nailing^10,21^. For the DTN samples, there is little to no pre-strain across the osteotomy, meaning that osteotomy completion will have left a gap of 0.5 mm (saw blade thickness) over which the proximal and distal fragments can move.

In all samples, the high strain zone in the lateral diaphysis is observed. It is not possible to know the location of the implants’ screws without CT data, but this area may correspond to strain from the screw exiting sites. Further analyses would need to be carried out to confirm this.

Nailing may be preferred over plating for vascular preservation reasons, and compression plating is often associated with soft tissue damage^14–16^. On the other hand, nailing also presents its limitations and has previously been associated with post-operative problems such as fragment malunion^29,30^. Concerning the DTN in particular, extensive reaming of the medial malleolus and distal epiphysis of the tibia for nail insertion may lead to necrosis and fracture of the distal tibia. We therefore recommend performing the opening of the entry portal and medullary canal with utmost caution. However, as this implant is still in early stages of use, no longitudinal studies has thus far been carried out and hence no post-operative complication highlighted.

The choice of implant is primarily based on the condition of the patient’s soft tissue envelope. In cases where vascularisation and soft tissues are already compromised, due to disease, age or previous injury, the use of a plate can lead to complications requiring a secondary surgery. In the distal tibia, problems such as skin irritation necrosis and infection have been reported^31,32^. The DTN may offer a solution to these problems: The implant is inserted in a minimally invasive way, only requiring small incisions at the tip of the medial malleolus and more proximally. In this way, the danger of as the potential skin irritation and necrosis zone is limited to the nail and screw insertion sites. The DTN is an implant designed specifically for the distal tibia and can cover a range of fracture and osteotomy zones^13^. Critical factors in using the DTN in retrograde tibial nailing are the selection of the correct entry point, placement of the guiding K-wire, low-pressure creation of the cavern through the medial malleolus with the crown reamer and subsequent low-pressure nail insertion^33^. In cases of posttraumatic malunions with completely obstructed intramedullary canal the use of a distal tibial nail might be limited or impossible. The potential risk of fracturing the medial malleolus must be taken into account^14^.

### Limitations

Sawbones® are composite bones that have been validated to imitate the mechanical properties of human bone^34,35^, resembling long bones of young adults. A medium sized Sawbones sample is based on the geometry of a 90-kg male of 1.83 m in height, less than 80 years of age^35^ and ensure that solely the bone-implant construct is evaluated and results are not influenced by bone quality, which can be a problem when using cadaver bones. However, this type of sample does not reflect reality and may conceal other problems related to both implants, such as with respect to vascularisation in plating and the possible fracture of the medial malleolus while developing the entry portal and the DTN insertion.

The inclusion of the fibula in the experimental setup is difficult to put in place; while 4th generation Sawbones composite fibula exist, there are currently no methods for considering the inter-osseous membrane that allows for tibio-fibular force transmission. We would expect that both axial and torsional stiffness levels would be higher in all samples with the addition of a fibula, with stiffness construct increasing by a similar level in all samples. This implies that a systematic error occurs in the omission of the fibula and should not be detrimental to the results.

No load-to-failure study was carried out for the samples; however, in a previous study by Kuhn et al.^10^, the DTN has shown superior biomechanical properties in load-to-failure tests for an A3 fracture configuration. In the given study, plastic deformation occurred in the plated samples between 350 and 500 N; complete fracture gap closure occurred at compressive loads between 500 and 700 N. In both cases, the DTN showed no signs of mechanical weakness.

Digital image correlation is a useful tool for the quantification of surface strain; however, it presents certain limitations. The facet size used for DIC was relatively high and may have resulted in the loss of information especially in the lateral cortex region. To improve this, it would have been necessary to create a finer speckle pattern on the samples. Extensometer and Von Mises’ measurements were taken at approximately the same area, but as the osteotomy and lateral cortex size were not controlled for and there are no anatomical landmarks in this zone, the placing of the extensometer and centre of the nodal disc in the Vic3D software is not entirely reproducible. In order to obtain DIC data for torsional testing, future studies may seek to concentrate on the lateral side of the tibia where the bone-bridge of the osteotomy is left. This would avoid any correlation problems due to an uneven surface caused by the presence of a plate. The use of an artificial landmark may provide a solution for DIC analysis and the positioning of inspection tools during the data processing procedure.

## Conclusion

In light of the results presented from construct stiffness and IFM in both best-case and worst-case scenarii, the DTN can be considered for SMOT fixation.

In the worst-case scenario, a significant difference is observed between the DTN and MDTP samples for the calculated compressive construct stiffness and the average ε_VM_ levels, which are greater and more concentrated in DTN samples than those observed in the MDTP samples. Despite this, fracture gap movement (IFM_mid_) of the DTN samples constantly remains inferior to 1 mm. Literature reports that fracture gaps of up to 1 mm are ideal for callous formation and bone healing^36–38^ and < 2 mm still promotes bone healing, but on a much slower scale than < 1 mm gaps, favouring fracture stability and fragment reunion^39^. In torsional testing, the DTN samples demonstrated greater resistance to the applied moments in most testing configurations; this is a favourable result as shear movements are known to be most harmful to fracture reconsolidation^24^.

## Data Availability

The datasets analysed during the current study are available from the corresponding author on reasonable request.
